# Combined Subcutaneous Fat Aspirate and Skin Tru-Cut Biopsy for Amyloid Screening in Patients with Suspected Systemic Amyloidosis

**DOI:** 10.3390/molecules26123649

**Published:** 2021-06-15

**Authors:** Charlotte Toftmann Hansen, Hanne E. H. Møller, Aleksandra Maria Rojek, Niels Marcussen, Hans Christian Beck, Niels Abildgaard

**Affiliations:** 1 Odense Amyloidosis Center, Odense University Hospital, 5000 Odense, Denmark; charlotte.toftmann.hansen2@rsyd.dk (C.T.H.); hanne.moeller@rsyd.dk (H.E.H.M.); aleksandra.maria.rojek@rsyd.dk (A.M.R.); niels.marcussen@rsyd.dk (N.M.); Hans.Christian.Beck@rsyd.dk (H.C.B.); 2 Department of Hematology, Odense University Hospital, 5000 Odense, Denmark; 3 Department of Pathology, Odense University Hospital, 5000 Odense, Denmark; 4 Department of Clinical Biochemistry and Pharmacology, Odense University Hospital, 5000 Odense, Denmark

**Keywords:** amyloid screening, fat aspirates, skin biopsies

## Abstract

Screening for systemic amyloidosis is typically carried out with abdominal fat aspirates with varying reported sensitivities. Fat aspirates are preferred for use in primary screening instead of organ biopsies as they are less invasive and thereby minimize the potential risk of complications. At Odense Amyloidosis Center, we performed a prospective study on whether the combined use of fat aspirate and tru-cut skin biopsy could increase the diagnostic sensitivity. Both fat aspirates and skin biopsies were screened with Congo Red staining, and positive biopsies were subsequently subtyped using immunoelectron microscopy and mass spectrometry. Seventy-six patients were included. In total, 24 patients had systemic amyloidosis (11 AL, 12 wtATTR, 1 AA), and 6 patients had localized amyloidosis. Combined fat aspirate and skin biopsy were Congo Red-positive in 15 patients (overall sensitivity (OS) 62.5%). Fat aspirates were positive in 14 patients (OS 58.3%), and the skin biopsy was positive in 5 patients (OS 20.8%). In only one patient did the skin biopsy add extra diagnostic information. The sensitivity differed between AL and ATTR amyloidosis—81.8% and 41.7%, respectively. Using skin biopsy as the only screening method is not recommended.

## 1. Introduction

Amyloidosis is the common name for a group of rare diseases with diverse symptoms and organ involvement caused by the extracellular deposition of different misfolded proteins. As many as 38 proteins have been identified as having amyloigenic potential, and even more are under investigation for being amyloigenic [1]. The most common types of systemic amyloidosis are immunoglobulin light chain (AL), mutant or wild type transthyretin (mATTR/wtATTR), reactive amyloid A (AA), fibrinogen (AFib) and apolipoprotein A-I (AApoAI). The treatment and follow-up of patients with amyloidosis depend on the involved protein, and therefore it is crucial to have sensitive and specific methods for amyloid identification and subtyping.

It is important to have a thorough diagnostic work-up for patients suspected of systemic amyloidosis. The diagnostic gold standard is to perform a biopsy of the involved organ, typically heart, liver or kidney. This method is, however, invasive and can be associated with complications, most frequently bleeding. Moreover, it is costly and requires specialist technical expertise and hospital equipment, which may introduce delay in the diagnostic phase. It has often proven possible to isolate amyloid from subcutaneous fat aspirates; a minimally invasive and low-cost procedure, with a low complication risk [2]. In addition, it is a simple procedure that can be performed bedside and without delay or further preparation of the patient.

Congo Red staining with its characteristic apple-green birefringence under polarized light is the current gold standard for identifying amyloid deposits in a biopsy [3]. An important step is to subtype the origin of the amyloid involved, since this will affect the following treatment options and follow-up of the patient.

Diagnostic subclassification has traditionally been challenging. Conventional immunohistochemistry (IHC) is problematic even in the most experienced laboratories, and has been abandoned in many pathology departments due to low sensitivity and specificity [4,5]. More sensitive techniques, such as mass spectrometry (MS) and immunoelectron (IEM) microscopy, have been incorporated in the diagnostic work-up at specialized amyloidosis centers. For the MS, proteins are proteolytically digested into peptides that are sequenced by tandem MS. Besides identifying the amyloid subtype, the method can identify a shared amyloid protein signature, which can be used as an internal positive quality control [6]. The extreme microscopic magnification of the IEM allows visualization of the amyloid fibrils. After immunostaining with gold-labeled specific antibodies, it can be visually assessed whether the antibodies specifically bind to the amyloid fibrils or nonspecifically bind to extracellular proteins not representing amyloid structures [7,8]. The combination of mass spectrometry (MS) and immunoelectron microscopy (IEM) has displayed very high sensitivity and specificity [8].

When screening for systemic amyloidosis with abdominal subcutaneous fat aspirates, specificities are uniformly reported as being high, while the diagnostic sensitivities are reported as being very variable, with values as low as 14% in some series and over 90% in others [9,10]. The sensitivity depends highly on the type of amyloid involved.

At Odense Amyloidosis Center, we performed a prospective study of whether the combined use of fat aspirates and simultaneously performed skin tru-cut biopsy could increase the diagnostic sensitivity.

## 2. Results

Seventy-six patients were screened for systemic amyloidosis with paired fat aspiration and skin tru-cut biopsy. Twenty-four patients turned out to have systemic amyloidosis, distributed as follows: two patients with AL-kappa, nine patients with AL-lambda, twelve patients with wtATTR and one patient with AA. The patient specific diagnostic findings in the 24 patients are presented in Table 1. All cases of systemic AL amyloidosis were verified by IEM and/or MS, and identification of B-cell clonality. ATTR cases were verified by IEM and/or MS, or by non-invasive established criteria including echocardiography and cardiac magnetic resonance imaging (CMR) findings combined with positivity in 3,3- diphosphono-1,2-propanodiacarboxylic acid (DPD)-scintigraphy [11,12]. In six patients, the work-up concluded that the patients had localized AL amyloidosis. Thus, in the remaining 46 patients, the diagnostic work-up did not reveal an amyloid diagnosis.

Of 24 patients with systemic amyloidosis, the combined use of fat aspirates and skin biopsy was Congo Red-positive in 15 patients, corresponding to an overall sensitivity of 62.5%, and a further diagnostic work-up by IEM and MS revealed the subtype. Of the 15 amyloid-positive combined biopsies, the fat aspirates were Congo Red-positive in 14 patients, equivalent to an overall sensitivity of 58.3%, and the skin biopsies were positive in 5 patients, equivalent to an overall sensitivity of 20.8% (Table 2). Thus, the skin biopsy performed significantly worse than fat aspirates (Fishers Exact test, *p* < 0.017), and added extra diagnostic information in only one patient, as the fat aspirate in this patient was negative.

The sensitivity of fat aspiration and skin biopsies was different for AL and ATTR. Of the 11 patients who turned out to have systemic AL amyloidosis, the combined use of both fat aspirate and skin biopsy gave rise to a sensitivity of 81.8%; although not statistically different in performance (Table 2), fat aspirate had a sensitivity of 72.7% versus a sensitivity of 36.4% for skin biopsy. Of the 12 patients with ATTR, the fat aspirate had a sensitivity of 41.7%, whereas none of the skin biopsies identified amyloid. In the patient with AA, both fat aspirate and skin biopsy were positive.

## 3. Discussion

The combined use of fat aspirates and skin tru-cut biopsies for amyloid screening showed an overall sensitivity of identifying systemic amyloidosis of 62.5%. A single use of fat aspiration yielded an overall sensitivity of 58.3%, which is comparable to the sensitivity reported by others [13,14].

The sensitivity of fat aspirate is highly dependent on which amyloid protein is involved. We found a sensitivity of 72.7% in patients with AL amyloidosis, but a lower sensitivity of 41.7% in patients with ATTR amyloidosis. Quarta et al. reported high sensitivities, especially in AL amyloidosis [13]. They also found the sensitivity to be dependent on the overall body amyloid burden. In our study, we did not take that aspect of the disease entity into account.

The techniques of the performed investigation, including amount of subcutaneous fat, number of blood vessels in the biopsy and contamination with blood in the smear, are important factors to bear in mind. Even though fat aspirate is a simple diagnostic procedure, it is important to be careful and thorough in the processing of the investigation [15].

Performing both skin biopsy and fat aspirate added diagnostic information in only 1 of 24 patients who were diagnosed with systemic amyloidosis.

In conclusion, abdominal fat aspiration is a simple, quick and reasonably sensitive diagnostic tool in the primary diagnostic work-up of patients with suspected systemic amyloidosis. Moreover, when positive for Congo Red amyloid deposits, it offers high diagnostic precision and specificity when combined with IEM and MS. Skin tru-cut biopsy performed significantly worse than fat aspirates, showing a lower sensitivity of only 20.8%, and it only added diagnostic information in one patient. Performing skin tru-cut biopsy as the only diagnostic procedure is not a reliable screening method in patients suspected of systemic amyloidosis.

## 4. Materials and Methods

Seventy-six patients suspected of systemic amyloidosis entered the screening program at Odense Amyloidosis Center, in the period from 1 January 2017 to 31 October 2019. Typical criteria for entering the screening program were suspicion of systemic amyloidosis based on, e.g., (1) identified abnormal free light kappa lambda ratio or M-component combined with proteinuria, elevated NT-pro brain natriuretic peptide, elevated liver function tests or unexplained weight loss; or (2) myocardial hypertrophia identified by echocardiography with characteristic co-findings, such as diastolic dysfunction and/or apical sparring; or (3) the identification of possibly localized amyloid in tissue biopsy. As part of the work-up, we performed simultaneous abdominal fat aspirates and skin tru-cut biopsies for Congo Red staining in all patients.

### 4.1. Skin Tru-Cut Biopsy

In the lower quadrant area of the abdominal wall, an area of 5 cm in diameter was cleansed and anesthetized. A 2 mm core biopsy was performed with a skin tru-cut biopsy, with special attention paid to the depth of the biopsy, to ensure the presence of subcutaneous tissue.

### 4.2. Fat Fine Needle Aspiration

The procedure was performed in the cleansed and anesthetized area as previously described by Gertz et al. [16]. We used an 18-gauge needle for the procedure. Fat tissue was removed from the syringe and placed on a glass slide. A second slide was placed on top of the first, and pressure was applied to crush the fat particles into a single cell layer. The specimens were allowed to air dry. Concomitantly, formalin-fixed fat tissue was stored for possible later IEM, and fresh frozen fat tissue kept for possible later MS.

### 4.3. Amyloid Identification and Sub-Classification by IEM and MS

Both fat aspirate imprints and skin biopsies were screened with Congo Red staining. Congo Red-positive biopsies were subsequently subtyped using IEM and MS as previously described [8] (see Supplementary Materials), with the following additions for fat aspirates where laser dissection microscopy of the amyloid deposits could not be performed. Fat aspirates were prepared for MS analysis by rinsing in 5 mL PBS three times, followed by proteolytic cleavage of the proteins by incubation in 20 µL 8 M urea with 0.5 µg lysC at 30 °C. After 4 h of incubation, samples were diluted to 1 M urea by addition of 80 0.2 M TEAB, and proteins were further proteolytically cleaved by incubation overnight at 30 °C in the presence of 0.5 µg trypsin.

All cases of AL amyloidosis were identified by IEM and/or MS in accordance with established criteria [8]. ATTR cases were identified by IEM and/or MS, or by established non-invasive criteria, as further described below.

### 4.4. Non-Invasive Identification of ATTR

Patients with suspected cardiac amyloidosis based on echocardiographic findings, e.g., concentric left ventricular thickening, severe impairment of diastolic function, restrictive Doppler filling patterns, severe impairment of longitudinal strain at the base of the left ventricle, with relatively well-preserved apical strain [17], were, in parallel with fat aspirate and skin biopsy, examined with cardiac magnetic resonance imaging (CMR) in most cases, and with 3,3- diphosphono-1,2-propanodiacarboxylic acid (DPD) scintigraphy in all cases. CMR-positive amyloid findings refer to characteristic global, left-ventricular late gadolinium enhancement and extracellular volume expansion on T1 mapping [11,12,17]. Due to pacemakers, implantable cardioverter defibrillators (ICD), or impaired kidney function not allowing the use of i.v. contrast agents, CMR could not be performed in a few patients. DPD-positive findings refer to the cardiac uptake of the tracer corresponding to a visual score of 2–3. DPD scintigraphy has proven a reliable tool for the detection of TTR-type cardiac amyloidosis (in the absence of a monoclonal gammopathy) [11,12].

## Figures and Tables

**Table 1:** Summary of diagnostic findings by Congo Red stain, immunoelectron microscopy analysis (IEM), mass spectrometry (MS), and complementary diagnostics.

Patient ID	Fat Aspirate			Skin Tru-Cut Biopsy			Complementary Diagnostic Workup	Diagnosis
	Congo Red Stain	IEM	MS	Congo Red Stain	IEM	MS		
1	+	Amyloid fibrils, kappa pos	Amyloid signature, plus kappa	+	-	-		AL-Kappa
2	+	No fibrils, negative	Amyloid signature, inconclusive	-	-	-	Echo: pos; DPD: pos; Clonality: no	ATTR
3	+	Amyloid fibrils, lambda pos	Amyloid signature, plus lambda	-	-	-		AL-Lambda
4	+	Amyloid fibrils, lambda pos	Amyloid signature, plus lambda	+	-	-		AL-Lambda
5	+	Amyloid fibrils, lambda pos	-	-	-	Amyloid signature, subtype inconclusive	BM: lambda clonal PC, amyloid (MS: amyloid signature, lambda).	AL-Lambda
6	-	-	-	-	-	-	Echo: pos; DPD: pos; BM: amyloid (IEM: no fibrils; MS: amyloid signature, ATTR)	ATTR
7	-	-	-	-	-	-	Echo: pos; BM: amyloid (IEM: amyloid fibrils, ATTR; MS: amyloid signature, ATTR)	ATTR
8	-	-	-	-	-	-	Echo: pos; DPD: pos; CMR: pos; Clonality: no	ATTR
9	-	-	-	-	-	-	Echo: pos; DPD: pos; CMR pos; Clonality: no	ATTR
10	-	-	-	-	-	-	Myocardial biopsy: amyloid (IEM: amyloid fibrils, AL-Lambda; MS: amyloid signature, AL-Lambda)	AL-Lambda
11	+	Amyloid fibrils, subtype inconclusive	No signature, negative	-	-	-	Echo: pos; DPD: pos; Clonality: no	ATTR
12	+	Amyloid fibrils, subtype inconclusive	No signature, negative	+	-	Amyloid signature, plus lambda		AL-Lambda
13	-	-	-	-	-	-	Echo: pos; DPD: pos; Clonality: no	ATTR
14	+	No fibrils, negative	No signature, negative	-	-	-	Echo: pos; DPD: pos; CMR pos; Clonality: no	ATTR
15	+	Amyloid fibrils, lambda pos	Amyloid signature, plus lambda	-	-	-		AL-Lambda
16	-	-	-	-	-	-	Lung biopsy: amyloid (MS amyloid signature plus lambda). Underlying Mb. Waldenström.	AL lambda
17	-	-	-	-	-	-	Echo: pos; DPD: pos; BM: amyloid (IEM: amyloid fibrils, ATTR; MS: amyloid signature, ATTR)	ATTR
18	-	-	-	-	-	-	Echo: pos; DPD: pos; CMR pos; Clonality: no	ATTR
19	+	Amyloid fibrils, lambda pos	Amyloid signature, plus lambda	-	-	-		AL-Lambda
20	+	Amyloid fibrils, kappa pos	Amyloid signature, plus kappa	+	-	-		AL-Kappa
21	+	No fibrils, negative	No signature, negative	-	-	-	Echo: pos; DPD: pos; Clonality: no	ATTR
22	+	Amyloid fibrils, Amyloid A pos	Amyloid signature, plus Amyloid A	+	-	-		AA amyloidosis
23	-	-	-	-	-	-	Lung biopsy: amyloid (IEM: AL-kappa; MS: amyloid signature, AL-kappa)	AL kappa
24	+	No fibrils, negative	Amyloid signature, inconclusive	-	-	-	Echo: pos; DPD: pos; CMR pos; BM without clonality or amyloid.	ATTR

Pos: positive; Echo: Echocardiography; DPD: 3,3-diphosphono-1,2-propanodiacarboxylic acid scintigraphy; CMR: cardiac magnetic resonance imaging; Clonality: no: normal serum free light kappa chain and free light lambda chain analysis, no M-protein in serum, and/or no B-cell clonality identified in bone marrow aspirate by flowcytometry; BM: bone marrow.

**Table 2:** The sensitivity and specificity of fat aspirate and skin biopsy, respectively.

	Fat Aspirate	Skin Biopsy	Fishers Exact Test (*p* Value)
Sensitivity	58.3%	20.8%	0.017
Sensitivity, AL	72.7%	36.4%	0.198
Sensitivity, ATTR	41.7%	0%	0.037
Specificity	88.5%	100%	

## Data Availability

All available results are presented in the text.

## Supplementary Materials

The following are available online, Figure S1: IEM images of amyloid patient samples, Table S1: MS data, Supplementary methods section.
