# Supplementary material for: Combined Subcutaneous Fat Aspirate and Skin Tru-Cut Biopsy for Amyloid Screening in Patients with Suspected Systemic Amyloidosis
Source: Molecules. 2021 Jun 15;26(12):3649. doi: 10.3390/molecules26123649 (PMC8232664; doi:10.3390/molecules26123649)
Supplement: Supplementary file 1 [file molecules-26-03649-s001.zip › Supplementary Figur 1_IEM images of amyloid patient samples.pdf]

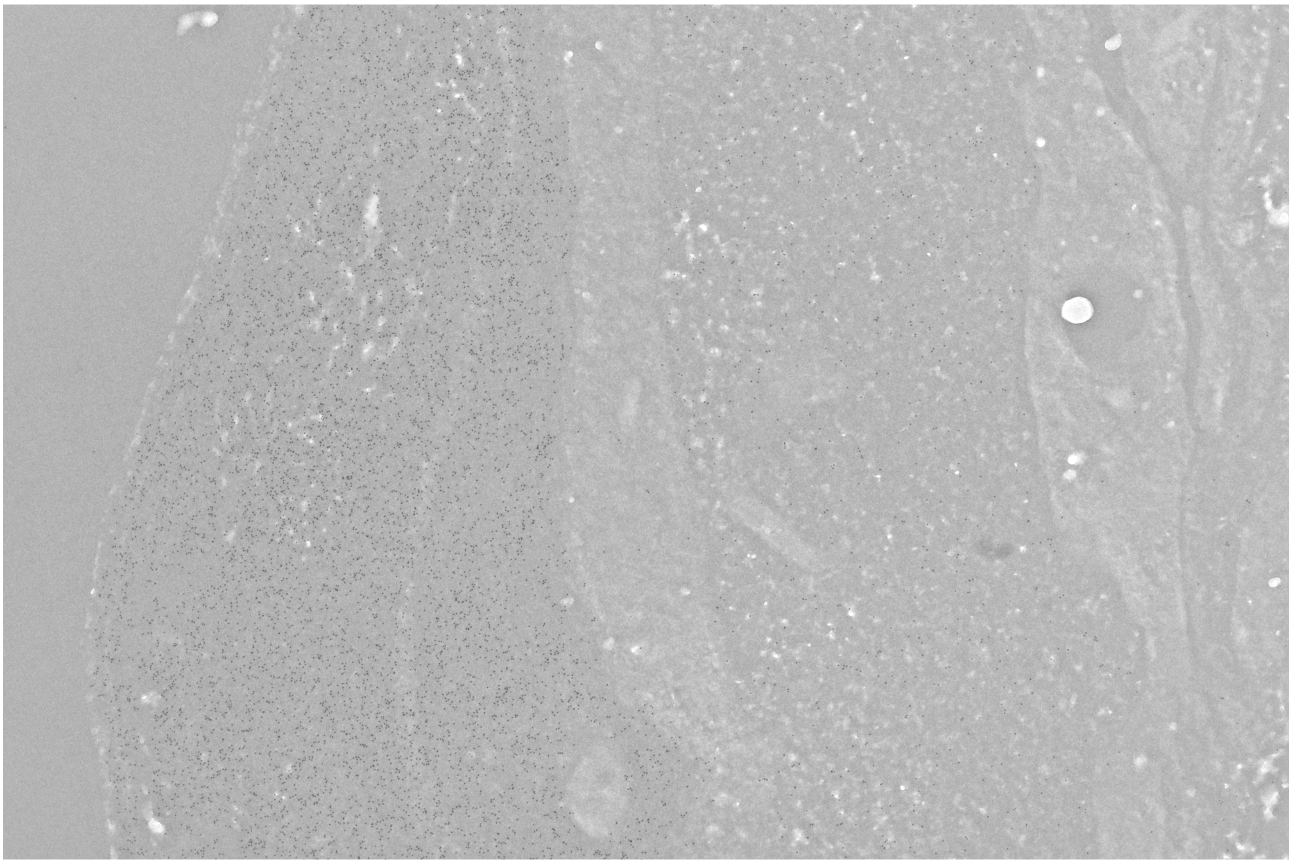

Patient 1. iTEM_46976 24, kappa

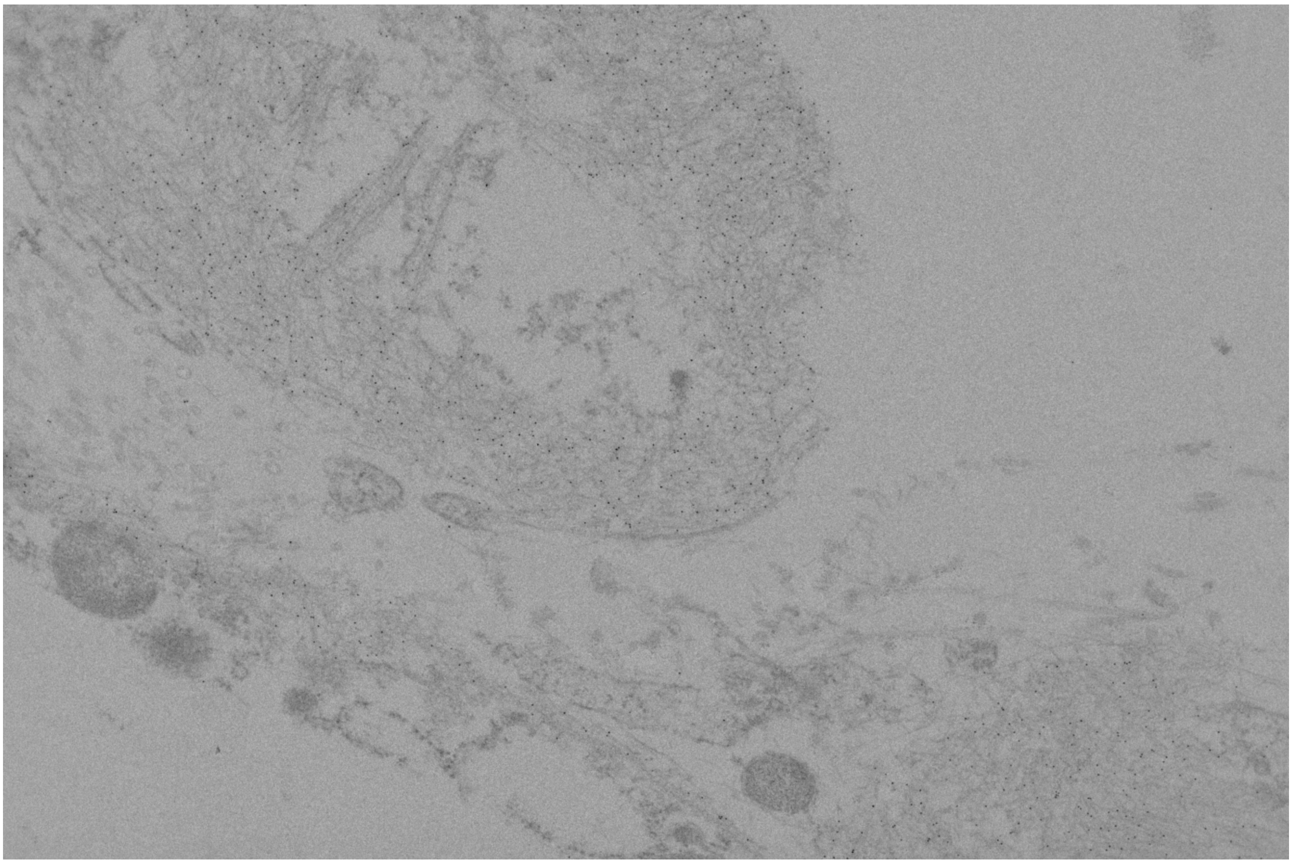

Patient 3. iTEM_61090 69, lambda

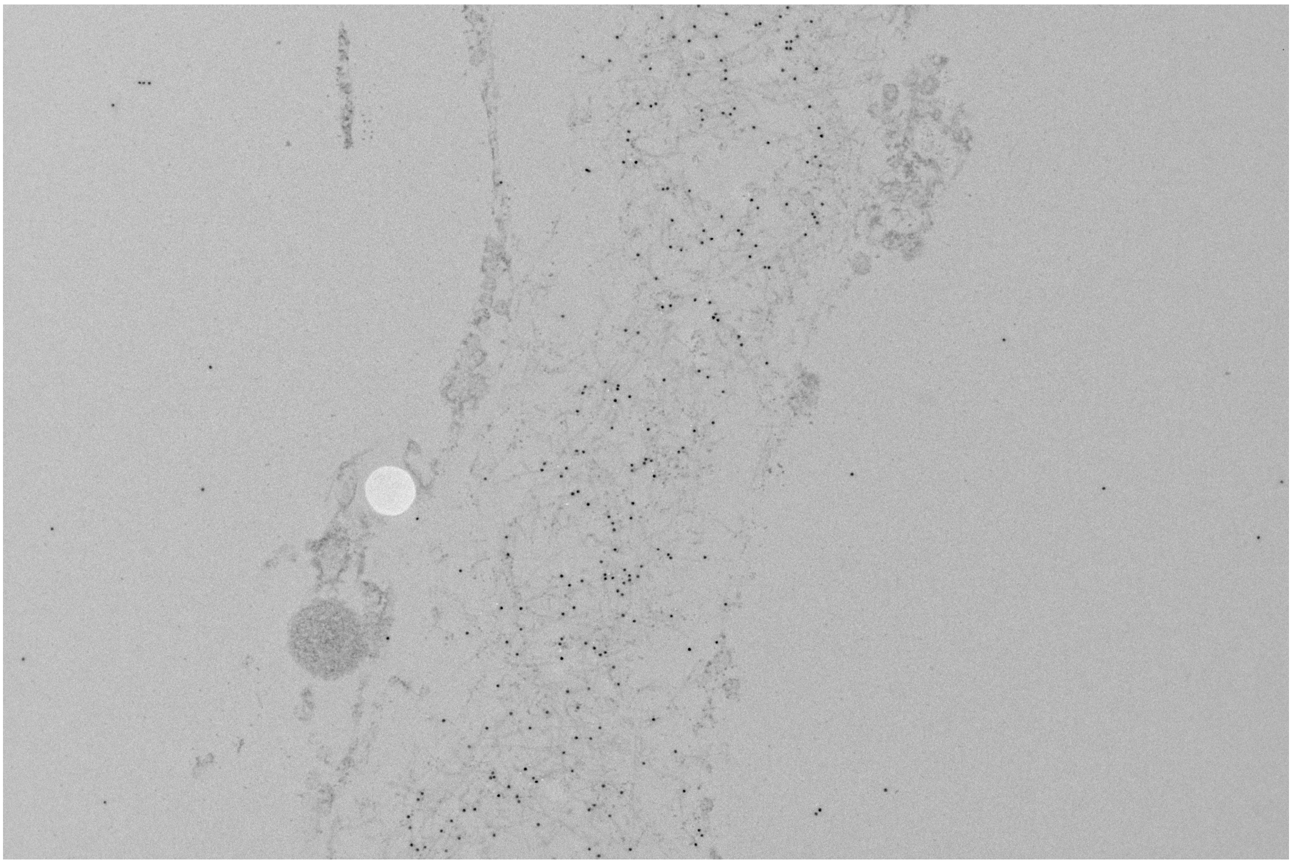

Patient 4. iTEM_50001 6, lambda

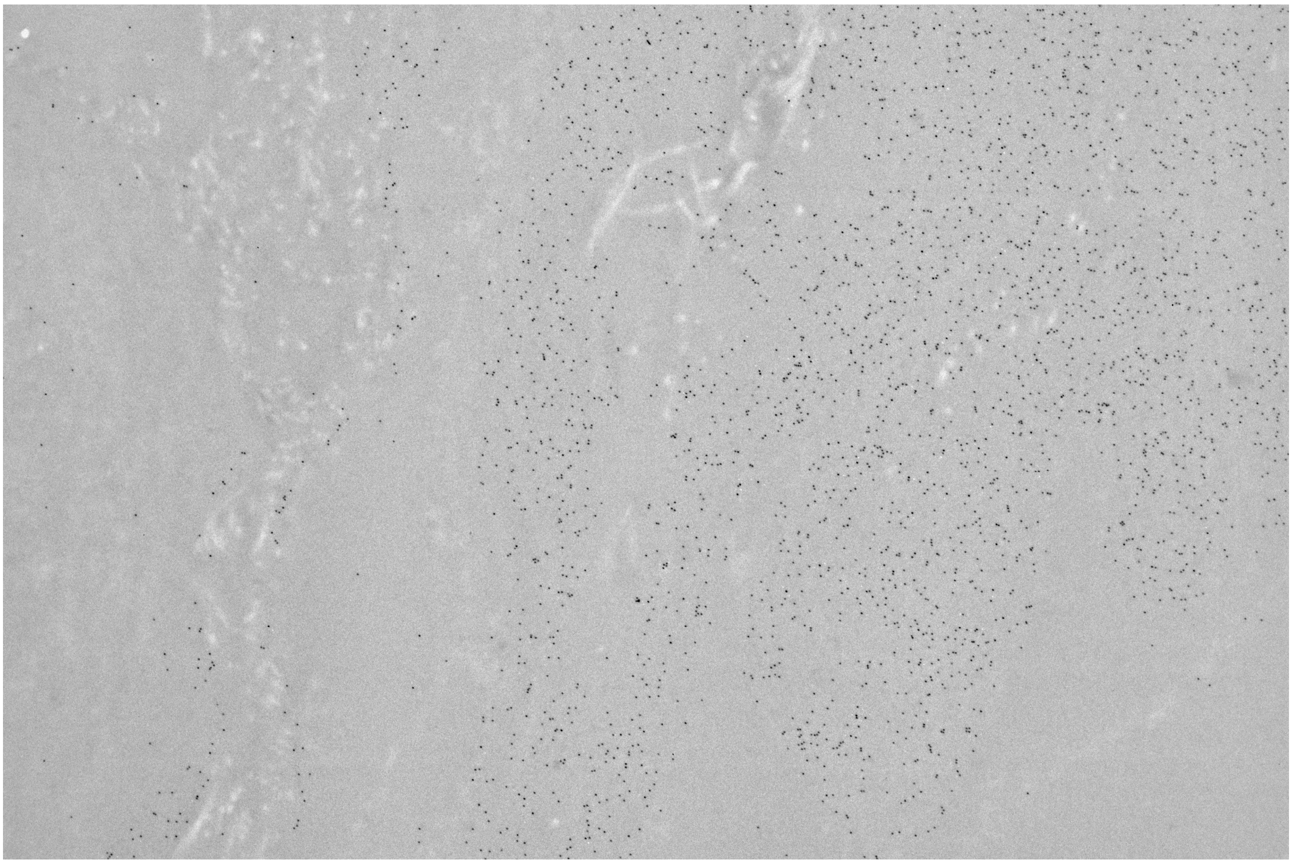

Patient 5. iTEM_43173 321, lambda

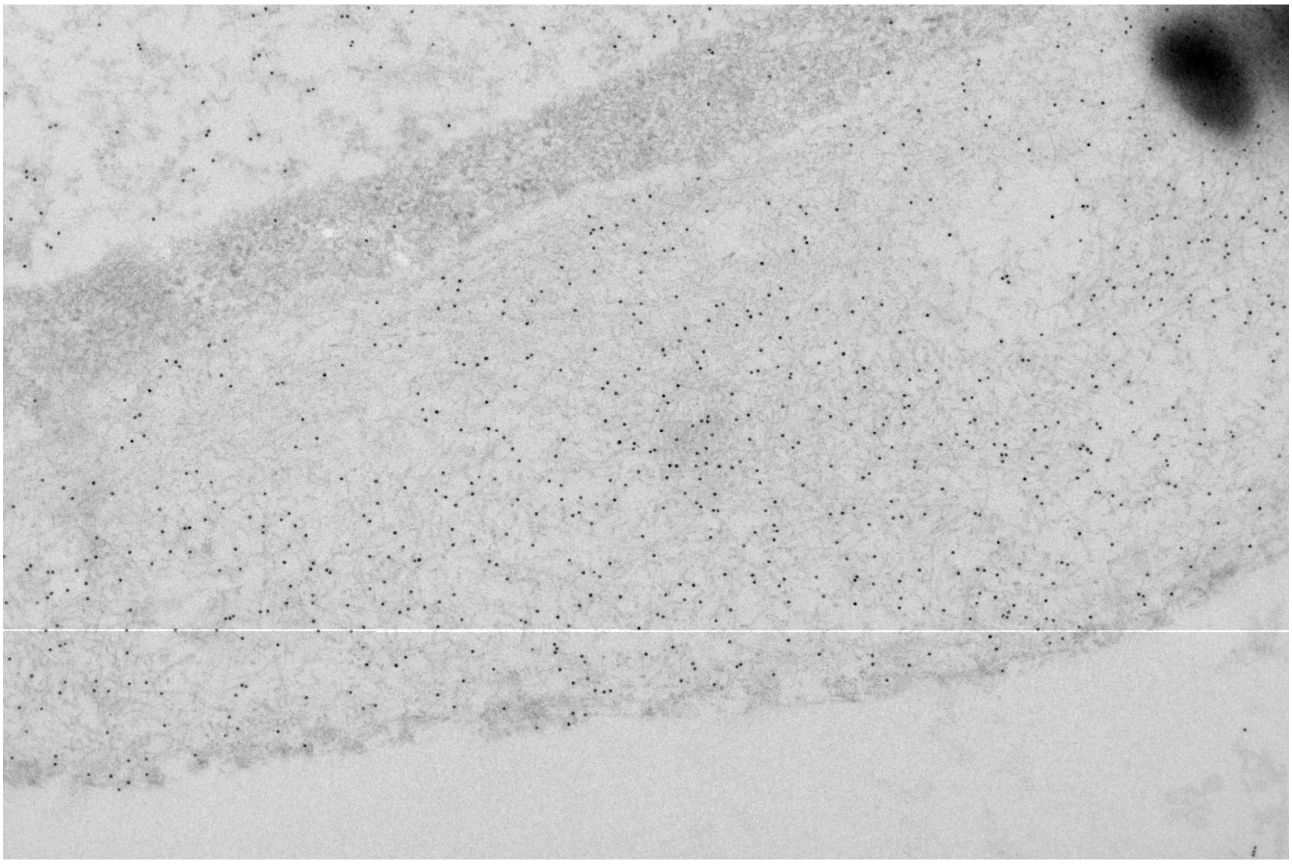

Patient 15. iTEM_9885 15, lambda

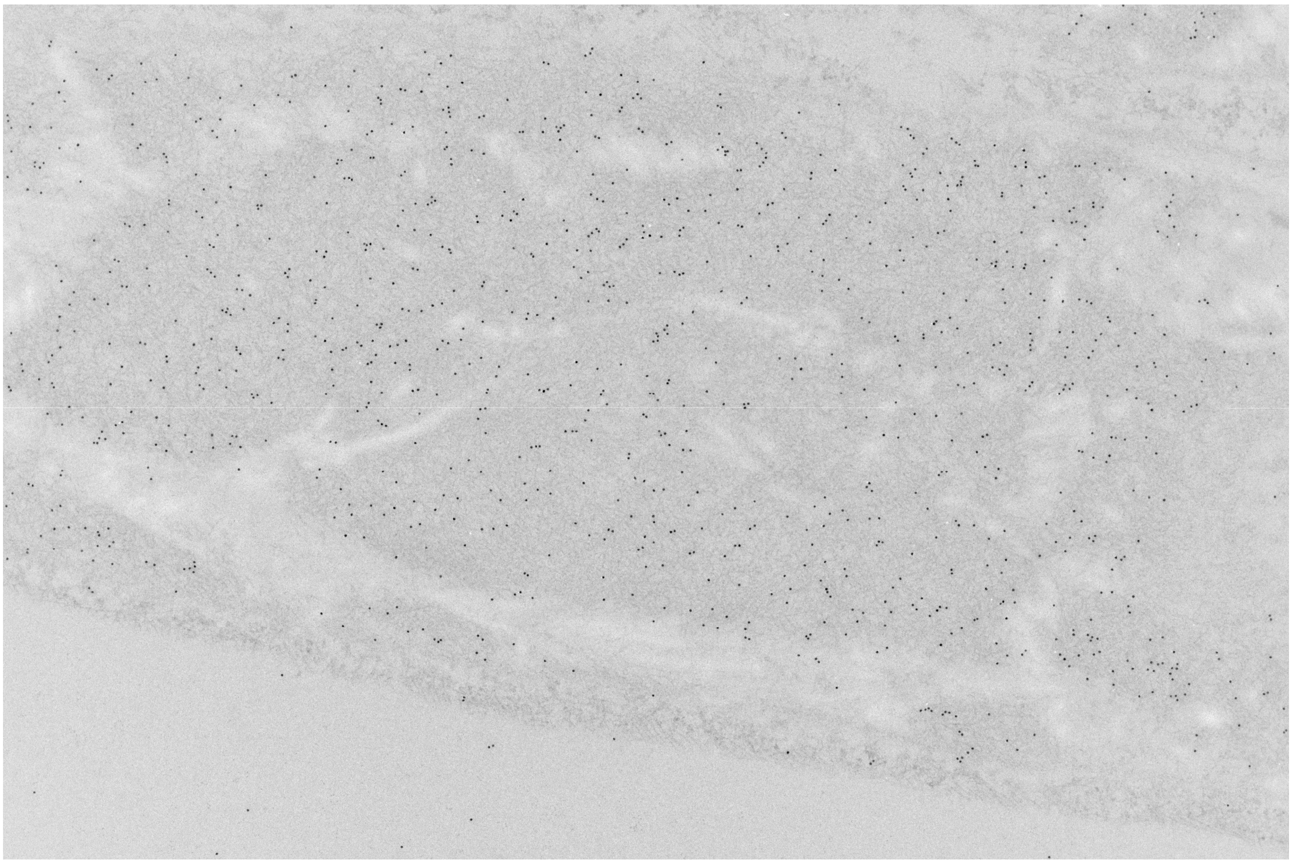

Patient 19. iTEM_57198 2, lambda

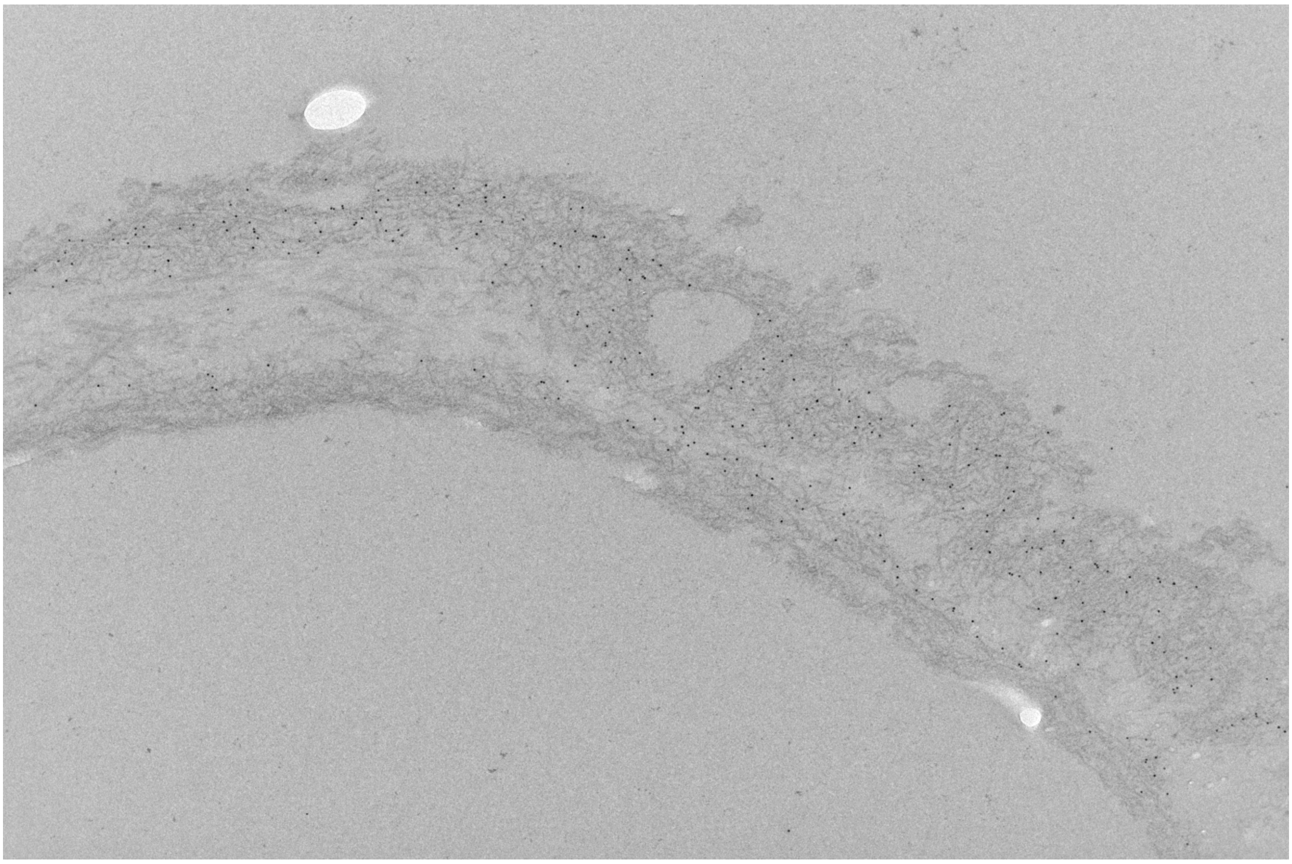

Patient 20. iTEM_61135 15, kappa

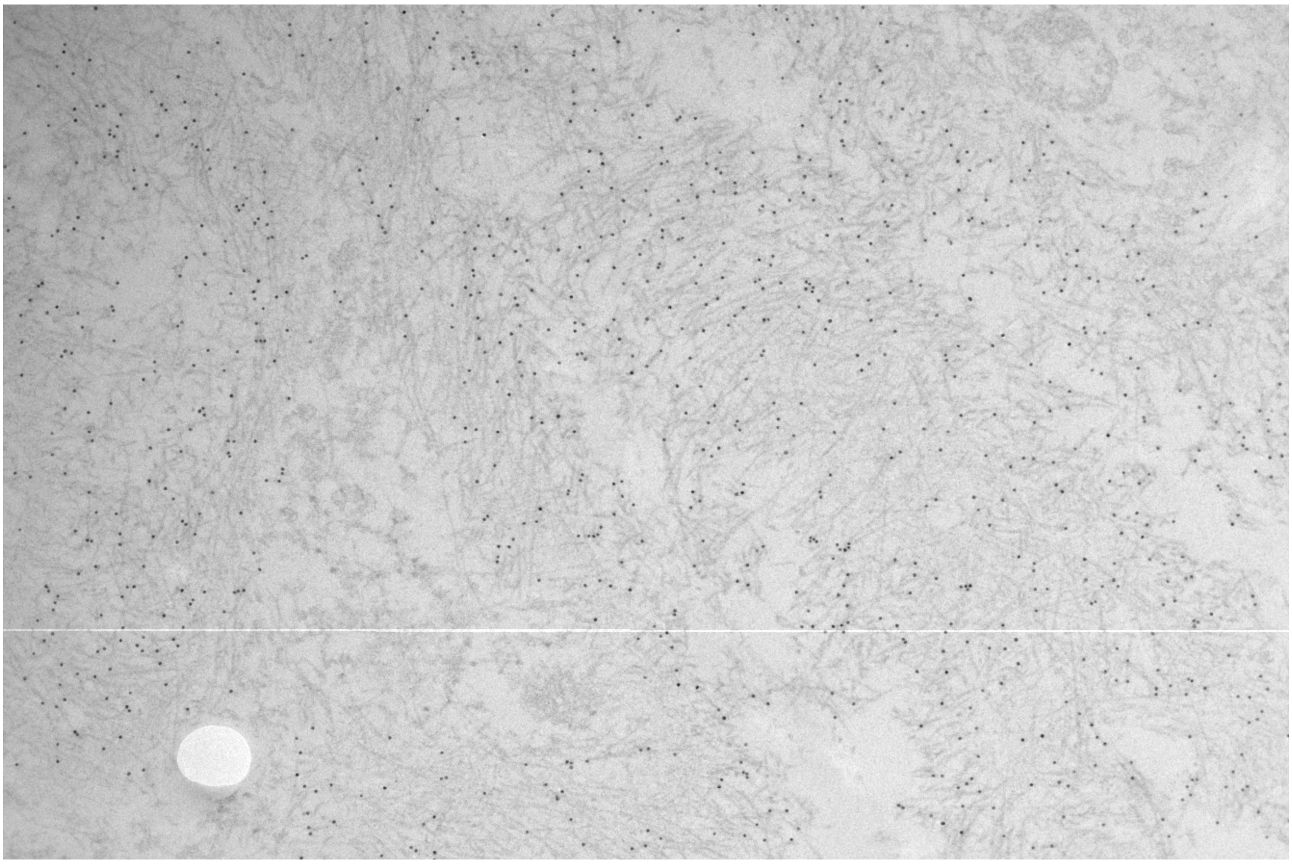

Patient 22. iTEM3733 25, amyA
