# Supplementary material for: Combined Subcutaneous Fat Aspirate and Skin Tru-Cut Biopsy for Amyloid Screening in Patients with Suspected Systemic Amyloidosis
Source: Molecules. 2021 Jun 15;26(12):3649. doi: 10.3390/molecules26123649 (PMC8232664; doi:10.3390/molecules26123649)
Supplement: Supplementary file 1 [file molecules-26-03649-s001.zip › Supplementary methods.pdf]

Immuno-electron microscopy and mass spectrometry

Immuno-electon microscopy

Preparation and processing of samples for IEM analysis and mass spectrometry analysis was done virtually as previously described (Abildgaard et al, 2020 (ref 8). Briefly, for IEM analysis, ultrathin (70 nm) sections stained with toluidine blue containing histological structures regions with potential amyloid deposits as identified by light microscopy were probed by the antibody of interest (anti-Serum amyloid A, anti-Kappa- and Lambda Ligth chains, and anti-Prealbumin (Transthyretin) followed by incubation with Protein A - 10 nm gold conjugate and visualization of amyloidogenic fibrils by electron microscopy. Immunostaining quality was validated by examination positive controls stained in parallel with the investigated samples. A positive finding demanded visual identification of distinct amyloid fibrillary structures and identified specific positive staining using gold-labelled antibodies against lambda, kappa, transthyretin, or serum amyloid A proteins.

Microdissection and sample processing

For laser dissection MS analysis 8 µm thick sections of formalin-fixed, paraffin-embedded (FFPE) patient specimens were fixated onto membrane slides, de-paraffinized, and Congo Red (CR) stained for visualization of amyloid deposits. Dissected areas (total area was approx. 0.2 mm^2) were prepared for proteome analysis as described in the previous study. Briefly, samples were incubated in 35 µl 10 mM Tris with 1 mM EDTA and 0,002% Zwittergent at 98° C for 90 minutes followed by reduction (50 mM DTT, 50° C, 30 min) and alkylation (150 mM IAA, RT in the dark, 30 min). Proteins were then acetone precipitated and re-dissolved in 20 µl, 200 mM triethylammonium bicarbonate (TEAB) followed by digestion, overnight, with 0.1 µg trypsin at 37° C. Purification of the resulting tryptic peptides were carried out using custom made C18 micro columns and the eluate was vacuum-centrifuged to dryness (SpeedVac, Thermo Scientific) and reconstituted in 0.1% TFA for analysis by nano-LC-MSMS, as described below.

Liquid Chromatography and Mass spectrometry

Tryptic digests were analysed by using an UltiMate3000 UHPLC unit coupled online to a Q-Exactive mass spectrometer fitted with a nano-electrospray ion source. Samples were loaded onto a custom-made, fused capillary pre-column (2 cm length, 360 µm OD, 75 µm ID packed with ReproSil Pur C18 3 µm resin (Dr. Maish, GmbH)) with a flow of 4 µl/min for 7 minutes. Trapped peptides were subsequently separated with a custom-made fuse capillary column (20 cm length, 360 µm OD, 100 µm ID, packed with ReproSil Pur C18 3 µm resin) employing a linear gradient from a 95 % solution A (0.1 % FA) toward a 28 % solution B (100 % acetonitrile in 0.1 % FA) over a 42-minutes interval with subsequent 5-minutes interval at 90 % B and 5-minutes 95 % A, with a flow rate of 0.3 µL/min. The Orbitrap MS scan was set to a target value of 1.000.000 ions at a resolution of 70.000 at m/z 200 and the MS/MS scan was set to a target value of 50.000 ions at a

resolution of 17.500 at m/z 200 (fixed first mass 110 m/z). Fragmentation occurred at a normalized collision energy of peptides in the HCD cell at 32 eV and intensity threshold for data dependent MSMS analysis was 27,000 counts/second.

Data analysis

All raw datafiles were processed using the Proteome Discoverer software (v. 2.4.0.305) and searched with the Sequest HT search algorithm. The search parameters were set to an MS accuracy of 8 ppm, MSMS accuracy of 0.05 Da for HCD data, with two missed cleavages allowed. Fixed modifications included carbamidomethylation at cysteine residues and variable modifications included methionine oxidation, deamidation of asparagine and glutamine and N-terminal acetylation. Raw datafiles were searched against the Swiss-Prot database restricted to the human proteome (downloaded on the 12th of December 2019, containing 20.303 entries). Proteins identified with at least one unique peptide and with a high confidence (FDR<1%) were permitted in the final dataset.

For all patient samples, the number of PSMs for the amyloid signature proteins (ApoA1, ApoA4, ApoE, and SAP) and the amyloid-associated proteins (most commonly TTR, AA, IG-K, and IG-K) were used to determine the true disease-state of a patient. The amyloid-associated protein with the highest number of PSMs were determined to be the pathogenic protein, prerequisite that at least 2 out of the 3 amyloid signature proteins were also detectable in the patient sample.
